# The relationship and mechanism between education and functional health status transition among older persons in China

**DOI:** 10.1186/s12877-018-0785-4

**Published:** 2018-04-11

**Authors:** He Chen, Hongwei Hu

**Affiliations:** 1 School of Public Health, Peking University, No.38, Xueyuan Road, Haidian District, 100191 Beijing, People’s Republic of China; 2 School of Public Administration and Policy, Renmin University of China, No. 59, Zhongguancun Street, Haidian District, 100872 Beijing, People’s Republic of China

**Keywords:** Education, Functional health status transition, Mortality, Disability, Transitional country

## Abstract

**Background:**

Despite decades of study, debates exist surrounding the relationship between education and functional health status transition among elderly populations. This study aims to add evidence to the debates using China as a case study. Specifically, this study analysed the association of education with functional health status transition and then the mechanism behind that association using the budget constraint relax hypothesis and the efficiency improvement hypothesis among elderly population in China.

**Methods:**

Based on data from the Chinese Longitudinal Healthy Longevity Surveys from 2008 and 2011, this study focussed on adults aged 65 years and above, with a final sample size of 12,112. A generalised structural equation model was used to analyse the relationship between education and functional health status transition and the mechanism behind that association.

**Results:**

During the three examined years, among elderly adults who were nondisabled at baseline, 53.1% stayed nondisabled, 14.6% became disabled, and 32.3% died; among those disabled in 2008, 8.1% recovered, 21.6% stayed disabled, and 70.3% died. Compared with older adults without any education, those who had attended primary schools had both lower mortality and disability, whereas those who had attended high schools and above only had a lower mortality rate. The budget constraint relax hypothesis and the efficiency improvement hypothesis explained the majority of the relationship between education and transition from non-disability to death, but hardly explained the transition from non-disability to disability. Furthermore, once a person was disabled, education had no significant relationship with functional ability recovery or mortality.

**Conclusions:**

Attending primary school seems to provide the highest benefit to functional health status transition among older and nondisabled persons in China. Those who attended high schools and above are expected to live a longer life with disability. The mechanism between education and the onset of disability needs more discussion.

## Background

Population is ageing worldwide due to declined mortality and fertility, with the majority of older persons living in under-developed and developing countries including China [1, 2]. Elderly health is essential to the sustainable development of an ageing society, while functional health status transition is one of the most important health indicators of elderly adults [3, 4]. Previous studies indicated that certain factors, including age, gender, chronic disease, mental health, socioeconomic status, education, and marriage, were associated with the functional health status transition of the elderly [5–7]. However, the specific roles certain factors play remain controversial; education is one such factor that arouses widespread debate. Education plays a fundamental and far-reaching role in the functional health status transition, and is regarded as a critical related factor [8]. However, previous studies concentrated mainly on certain specific aspects of the association and the pathways between education and functional health status transition, which limited the overall understanding of the actual role of education in functional health status transition.

Most studies have indicated that there was a protective effect of education on functional health status transition, and elderly people with higher education levels had a lower probability of mortality and a higher probability of recovery from functional disability [9, 10]. One study showed that elderly adults who had nine or more years of education were less likely to have disability compared with their counterparts [11]. Another study indicated that individual functional health status decreased with the improvement of education. The Activities of Daily Livings (ADLs, a physical health indicator measuring individuals’ ability to perform daily activities, such as walking, bathing, grooming, dressing, eating, transferring, and toileting) score of the elderly decreased by 0.03 to 0.04 as their years of education increased by 1 year [12]. In addition, studies have indicated that education remained significantly associated with the development of functional health status over time [13, 14].

On the other hand, some studies have found an inconsistent relationship between education and functional health status transition, indicating that there was little effect of education level on the reduction of functional disability [15–17]. Among elderly women, education has been shown to have a tendency to reduce mortality, but not to be significantly associated with disability-transition [18, 19]. The significance of association between education and functional health status transition among the elderly disappeared when the data were adjusted for confounding variables [20].

Some previous studies also focussed on the pathway and mechanism between education and functional health status transition. Budget constraint relax hypothesis and efficiency improvement hypothesis were developed based on accumulated previous studies to explain the mechanism between education attainment and functional health status transition [21, 22]. According to studies that reflected the budget constraint relax hypothesis, higher education attainment played a protective role in reducing the probability of disability and mortality by promoting one’s capability of acquiring health-related resources, including better jobs, higher salary, better health insurance, and services [23–25]. In some studies, while controlling for different health-related resources as mediators, the significance of the association between education and functional health status transition was reduced, but was present to a varying degree [26, 27]. According to other research that reflected the efficiency improvement hypothesis, higher education attainment may promote one’s productive efficiency in producing health outcomes. Educated elderly people were more likely to have the wisdom to allocate health-related resources and to adopt a healthy lifestyle (regular exercise, balanced diet, etc.), and the significance of association between education and functional health status transition decreased in varying degrees when controlling for health-related knowledge and behaviours [28–33]. In short, the significance of the association between education and functional health status transition decreased or disappeared while controlling for a series of variables according to the two hypotheses.

Previous studies deepened the understanding of the association and mechanism between education and functional health status transition. However, several limitations exist. First, the majority of previous studies investigated the association between education and disability or death separately among the elderly rather than looking at an integrated-pattern transition from disability to death, which failed to illustrate the comprehensive relationship between education and functional health status transition. Second, most previous studies explored some specific factors rather than researching a systematic mechanism including all the potential factors as mediators to explain the connection between education and functional health status transition, leaving this topic incomplete. Third, there is a lack of research concerning Chinese elderly people; more related research is needed.

To provide more evidence on the role of education in elderly health, this study aims to investigate the association between education and functional health status transition and analyse its mechanism using the budget constraint relax hypothesis and the efficiency improvement hypothesis among the elderly in China on the basis of longitudinal data. In particular, the generalised structural equation model (GSEM) was used in this study to test the two hypotheses with a series of variables as mediators, and coefficients of regression from the GSEM were shown to estimate the mediating effects of all the variables. The change of estimation and significance of the parameters from the GSEM can help us to identify and justify the two hypotheses.

## Methods

### Conceptual framework

**Fig. 1:**
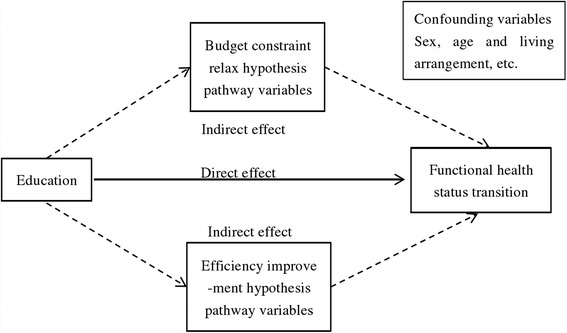
Conceptual framework on the relationship between education and functional health status transition

Education is a critical predisposing and socioeconomic factor that exerts far-reaching influence on functional health status transitions in one’s late life. Variation in functional health status transition among elderly adults is partly attributable to different education levels. In this study, both the budget constraint relax hypothesis and the efficiency improvement hypothesis were employed to analyse the pathway between education and functional health status transition of elderly adults using a series of variables. More details of these two hypotheses are presented below. The whole conceptual framework is demonstrated in Fig. 1.

### Source of data

**Fig. 2:**
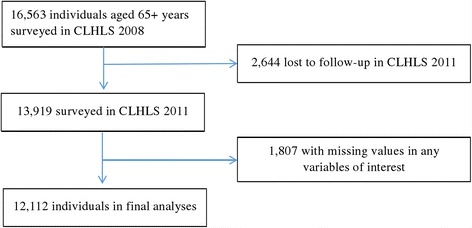
Flowchart of participants used for analyses

The Chinese Longitudinal Healthy Longevity Survey (CLHLS) aims to shed light on the determinants of healthy human longevity. It collects information on health, socioeconomic characteristics, family, lifestyle, and the demographic profile of the elderly aged 65 and above in China [34]. The survey has been conducted every 3 years in six waves, from 1998 to 2011, in 631 randomly selected counties in China’s 31 provinces [35]. The data from the CLHLS are of high quality according to its representativeness and randomness of attrition [36, 37]. Longitudinal data used in this study were from the 2008 fifth wave and the 2011 sixth wave of the CLHLS. A total of 12,112 participants that provided followed up responses from 2008 to 2011 with complete information on key variables were used in this study (Fig. 2).

### Measurement

#### Functional health status transition

This paper analysed the functional health status transition for Chinese elderly people from 2008 to 2011. The functional health status was divided into three types: nondisabled, disabled, and death. Disability was measured with ADLs. Those who needed help finishing any of the following items were regarded as disabled: bathing, dressing, toileting, indoor transferring, continence, and eating. For those who were nondisabled at the baseline, we created ‘transition1’ with three types of transition: (1) nondisabled (2008) to nondisabled (2011); (2) nondisabled to disabled; (3) nondisabled to death. For those who were disabled at baseline, we created ‘transition2’ with three types of transition: (1) disabled to nondisabled; (2) disabled to disabled; (3) disabled to death.

#### Education

CLHLS 2008 asked about school years that respondents attended. Considering the education setting in China, the education attainment of the final sample is as follows: 7628 persons (62.98%) did not receive any education (0 years), 3349 (27.64%) attended some primary school (1–6 years), 621 (5.13%) attended some junior high school (7–9 years), 352 (2.91%) attended senior high school (10–12 years), and only 162 (1.35%) attended college and above (at least 13 years). The number of respondents who had ever received an education of junior high school level and above was small; therefore, a broader education level with three categories was chosen in this study in order to obtain robust results: no school (0 years), primary school (1–6 years), and high school and above (at least 7 years).

#### Budget constraint relax hypothesis

According to the budget constraint relax hypothesis, education attainment may expand the resources one can invest in health. To examine this hypothesis, we included respondents’ characteristics measured at baseline. (1) Poor (1 = yes, 0 = no). CLHLS asked the respondents to ‘Rate your economic status compared with others in your local area’. Six options were provided: very rich, rich, so-so, poor, very poor, and no answer. We combined the options of poor and very poor into ‘yes’, very rich, rich and so-so into ‘no’, and recoded ‘no answer’ into missing values. (2) Professional/management occupation before 60 years old (1 = yes, 0 = no). CLHLS asked respondents about their primary occupation before age 60. Eight options were provided: professional or technical personnel/doctor/teacher; staff/service worker/industrial worker; self-employed; agriculture, forestry, animal husbandry, fishery; housework; military personnel; unemployed; other. We created the variable of ‘professional/management occupation before 60 years old’ with the first option as ‘yes’ and all other options as ‘no’. (3) House ownership (1 = yes, 0 = no). If the house that a respondent lived in was purchased, self-built, inherited, or welfare-oriented public housing, the respondent was regarded as owning the house. (4) Economic independence (1 = yes, 0 = no). CLHLS asked for the source of respondents’ primary financial support with eight options including retirement wages, spouse, child(ren), grandchild(ren), relative(s), local government or community, work salary and other sources. We recoded ‘retirement wages’ and ‘work salary’ into ‘yes’ and other options into ‘no.’

#### Efficiency improvement hypothesis

According to efficiency improvement hypothesis, education may promote health by enhancing productive and allocative efficiency in health-related resources. To examine this hypothesis, we selected four variables measured at baseline. (1) Regular exercise (1 = yes, 0 = no); (2) Balanced diet (i.e. eating fresh fruits or vegetables quite often or almost every day and eating meat, fish, eggs, bean products, or milk products at least once per week or almost every day. 1 = yes, 0 = no). (3) Leisure activities including gardening work, reading newspapers or books, playing cards or mah-jongg, watching TV or listening to the radio, and taking part in social activities. Each item had five options: nearly every day, at least once per week, at least once per month, sometimes, and never. These options were scored ranging from 1 (nearly every day) to 5 (never). The respondents with a total score below the mean value of 21.2 were regarded as having a high level of leisure activities; otherwise, low leisure activities. (4) Overweight or obesity was defined as having a body mass index (BMI = kg/m^2^) equal to or higher than 24.

#### Control variables

Control variables included demographics, living arrangements, and parental socioeconomic variables. (1) Demographic variables included age groups (1 = 65–69, 2 = 70–74, 3 = 75–79, 4 = 80–84, 5 = 85–89, 6 = 90 and above), gender (1 = male; 0 = female), and residence (1 = urban; 0 = rural). (2) Living arrangement variables included living with a spouse (1 = yes, 0 = no) and living with children (1 = yes, 0 = no). (3) Parental socioeconomic variables included whether the participant’s father had ever received an education (1 = yes, 0 = no), father’s occupation before 60 years old (1 = professional/management; 0 = no), whether respondents suffered from hunger during childhood (1 = yes, 0 = no), and respondent’s birth place (1 = urban, 0 = rural).

### Analyses

We used frequency and percentage to describe sample characteristics. A generalised structural equation model was used to analyse the relationship between education and functional health status transition as well as the mediating effects of variables using the budget constraint relax hypothesis and the efficiency improvement hypothesis. Binary outcome variables are from the Bernoulli family and were analysed using logit link; categorical outcome variables are from multinomial family and were analysed using logit link. The maximum likelihood method was used to estimate the parameters. All the analyses were performed with Stata 15.1.

To analyse the relationship and mechanism between education and functional health status transition among older persons in China, we carried out three generalised structural equation models. First, Model 1 included transition1 and transition2 working as outcome variables and education as explanatory variables. In the multinomial logistic regression of transition1, the type of ‘nondisabled-nondisabled’ was the base outcome; in the multinomial logistic regression of transition2, the type of ‘disabled-nondisabled’ was the base outcome; the education level of no schooling was the reference category. The Akaike and Bayesian information criteria (AIC and BIC) values for Model 1 were 22,564.31 and 22,653.14, respectively. Second, Model 2 was specified as follows: (1) transition1 and transition2 were outcome variables with education, demographic, living arrangement, and parental socioeconomic variables as explanatory variables; (2) education was the outcome variable with demographic and parental socioeconomic variables as explanatory variables; and (3) other specification was the same as in Model 1. AIC and BIC values for Model 2 were 20,048.17 and 20,521.9, respectively. Third, Model 3 was specified as follows: (1) Budget constraint relax hypothesis variables (poor, professional/management occupation before 60 years old, house ownership, and economic independence) and efficiency improvement hypothesis variables (regular exercise, balanced diet, high leisure activities, and overweight or obese) were outcome variables with education, demographic, living arrangement, and parental socioeconomic variables as explanatory variables; (2) transition1 and transition2 were outcome variables with education, budget constraint relax hypothesis variables, efficiency improvement hypothesis variables, demographics, living arrangement, and parental socioeconomic variables as explanatory variables; and (3) other specification was the same as in Model 2. AIC and BIC values for Model 3 were 111,814.4 and 113,650.1, respectively.

## Results

Among participants, 72.03% were aged 80 years old and above, 43.63% were male, 35.34% lived in an urban area, 31.61% lived with spouses, and 58.45% lived with children. In addition, 75.89% of subjects had ever suffered from hunger due to lack of food during childhood, and 10.51% were born in urban areas. The majority (62.98%) did not have any education, 27.65% received some primary education, and 9.37% received an education of high school and above. More information is presented in Table 1.

**Table 1:** Sample characteristics (*n* = 12,112)

Variables	Number	Percent
Socio-demographics		
Age groups		
65–69	1077	8.89
70–74	1220	10.07
75–79	1090	9.00
80–84	1509	12.46
85–89	1712	14.13
90 and above	5504	45.44
Male	5285	43.63
Urban residence	4280	35.34
Living with spouse	3829	31.61
Living with children	7079	58.45
Father with education	2026	16.73
Father with professional/ management occupation	280	2.31
Suffering from hunger during childhood	9192	75.89
Born in urban areas	1273	10.51
Education		
No school	7628	62.98
Primary school	3349	27.65
High school and above	1135	9.37
Budget constraint relax hypothesis variables		
Poor	2217	18.30
Professional/management occupation before 60 years old	775	6.40
House ownership	5050	41.69
Economic independence	2929	24.18
Efficiency improvement hypothesis variables		
Regular exercise	3291	27.17
Balanced diet	432	3.57
High level of leisure activities	6482	53.52
Overweight or obesity	1626	13.42
Functional health status transition		
For those nondisabled in 2008 (n = 9805)		
Nondisabled to nondisabled	5208	53.12
Nondisabled to disabled	1435	14.64
Nondisabled to death	3162	32.25
For those disabled in 2008 (*n* = 2307)		
Disabled to nondisabled	187	8.11
Disabled to disabled	499	21.63
Disabled to death	1621	70.26

Among subjects who were nondisabled in 2008 (*n* = 9805), more than half were still nondisabled, 14.64% became disabled, and nearly 1/3 were dead 3 years later. On the other hand, among those disabled in 2008, less than 10% recovered their abilities to carry out daily activities, 21.63% were still disabled, and 70.26% were dead 3 years later.

**Fig. 3:**
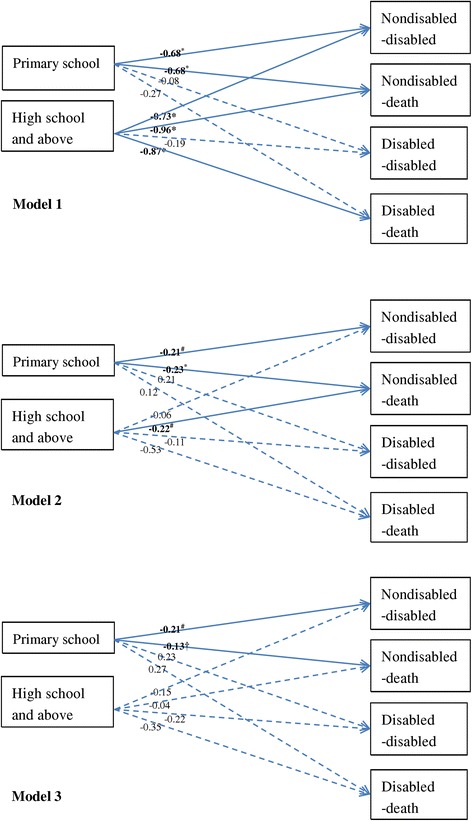
Coefficients of education in the multinomial logistic regression of functional health status transition in Models 1–3

The coefficients of education in Models 1, 2, and 3 are displayed in Fig. 3. In Model 1, compared with those without any schooling, educated older persons had a significant advantage in avoiding disability and death for those nondisabled at baseline; older persons who had attended high school and above had a significantly lower probability of death for those disabled at baseline during the 3 years. After bringing demographics, living arrangements, and parental socioeconomic variables into Model 2, the relationship between education and functional health status transition weakened: the effect of primary school on avoiding disability and death for those nondisabled at the baseline decreased; attending high school and above was only significantly and negatively associated with a probability of death for those nondisabled at baseline. Furthermore, compared with the results in Model 2, the benefits of education on mortality largely weakened or even disappeared among nondisabled older persons in Model 3: the coefficient of primary school changed from − 0.23 (*p* < 0.01) to − 0.13 (*p* < 0.10), and the coefficient of high school and above changed from − 0.22 (*p* < 0.05) to − 0.04 (*p* > 0.10) (Fig. 3).

**Fig. 4:**
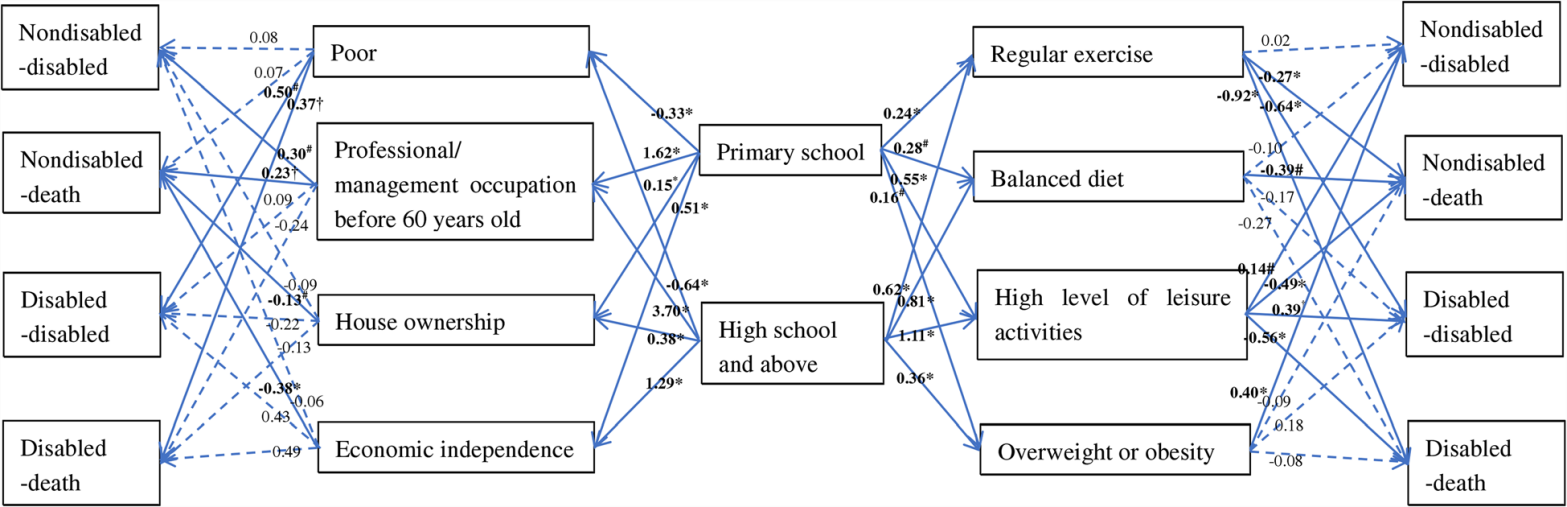
The coefficients of the budget constraint relax hypothesis and the efficiency improvement hypothesis variables in the regression of the functional health status transition in Model 3

The changes in the relationship between education and functional health status transition between Model 2 and Model 3 could be explained by the mediating effects of the budget constraint relax hypothesis and the efficiency improvement hypothesis variables (Fig. 4). Compared with no schooling, education significantly increased the probability of house ownership, economic independence, regular exercise, balanced diet, and high leisure activities, which significantly decreased mortality among nondisabled older persons. At the same time, education increased the probability of having a professional or management occupation before retirement, which significantly increased the mortality rate among nondisabled older persons. Moreover, the relationship between primary education and disability prevention did not change much between Models 2 and 3. The effect of mediating variables was mixed. Compared to those without any education, older persons with a primary education were more likely to have a professional/management job and to be overweight or obese, which turned to an increase in the risk of losing their abilities of doing daily activities. On the other hand, older persons with primary educations engaged in more leisure activities, which helped them prevent disability.

Additionally, even though the results of Models 2 and 3 did not show a direct effect of education on functional health status transition for disabled older persons, education probably enhanced functional health recovery by improving income and increasing regular exercise, and prevented death by increasing leisure activities.

## Discussion

Using longitudinal CLHLS data (2008–2011), we found a complicated relationship between education and functional health status transition and verified the applicability of the budget constraint relax hypothesis and the efficiency improvement hypothesis in explaining it. This study added evidence to the debate around the protective effect of education on functional health status transition and further deepened the understanding of the relationship by analysing its mechanism.

For an older person who was nondisabled at baseline, different levels of educational attainments had varying relationships with the functional health status transition. Compared with elderly persons without any education, those who had ever attended primary school had a significant advantage in delaying the onset of death or disability; however, those who had ever attended high school and above benefitted only on delaying death, not disability, which resulted in a longer life expectancy (living with a disability).

Despite the fact that most previous studies showed a gradient association of education attainment with functional health status transition, some researchers found little impact of schooling on the onset of disability or death [38, 39]. Our results indicate that there is a necessity of dividing education into smaller categories instead of dummy or continuous variables, which may provide more insight into the relationship [40, 41]. In addition, the majority of studies on this topic were conducted in developed countries, and this study was conducted in China, a developing country in the midst of a profound health transition [42]. The different socio-economic and health environments of the study population may also exert an influence on the conclusions.

Another contribution of this present paper was to explore the mechanism between education and functional health status transition by testing both the budget constraint relax hypothesis and the efficiency improvement hypothesis. Both hypotheses minimally explained the relationship between education and transition from non-disability to disability. Although older persons with higher education levels tended to have advantages with all mediating variables except overweight or obese, only a few variables had a significant relationship with the onset of disability among elderly persons who were nondisabled at baseline. This finding further deepened the understanding of the details about the mediating effects mentioned in previous studies, which showed that education exerted significant influence on promoting health and preventing disability in part through these mediating variables [43–46]. On the other hand, both hypotheses exhibited a strong power to explain the relationship between education and the transition from non-disability to death. Mediating variables including house ownership, economic independence, regular exercise, balanced diet, and a high level of leisure activities were related to reduced mortality. Several studies have shown that occupation is associated with mortality risk, and different occupation types are likely to increase or decrease the mortality risk [47–49]. Some sedentary occupation types, such as management and professional types, were associated with higher mortality risk, and this relationship was also found in the present study [50].

This study showed that, once elderly adults became disabled, they probably no longer benefitted from education in terms of recovering from disability or preventing death. Our findings contradict studies indicating that disabled elderly adults with higher education levels were more likely to recover from disability [11, 51]. On the other hand, some researchers have found similar results as ours, showing that education only delayed the onset of disability and did not affect functional recovery once elderly adults became disabled [28, 42, 52, 53].

There are some limitations of this present paper. First, there was only a 3-year interval between the CLHLS 2008 and 2011 waves, which may limit our capability to discover the longer-term impact of education on functional health status transition. Second, due to data availability, we included eight mediating variables to test the budget constraint relax hypothesis and the efficiency improvement hypothesis, which may partly affect the verification of the applicability of these two hypotheses in explaining the relationship between education and health transition. Third, CLHLS had a 3-year interval between the two waves of data used in this study, and did not collect participants’ information on functional status transition between the intermediate years. This possibly led to some bias in our study.

## Conclusions

This present paper deepened the understanding about the complicated role of education on functional health status transition. Disparities exist in functional health status transition across nondisabled older persons with different levels of education. Those attending primary school seemed to benefit the most from education, with both lower mortality and disability risks, whereas those attending high schools and above only had a lower mortality risk but a similar disability level with their counterparts without any schooling. In other words, those attending high schools and above were expected to live a longer life with disability. In addition, the budget constraint relax hypothesis variables, including house ownership and economic independence, and the efficiency improvement hypothesis variables, including regular exercise, balanced diet, and high level of leisure activities, were the main mediating factors between education and mortality. However, these two hypotheses minimally explained the relationship between education and disability, which needs further discussion in future studies. Moreover, once older persons became disabled, education had no significant relationship with their functional ability recovery or the onset of mortality.

The study has at least the following implications for policymaking. In addition to older persons with no schooling, Chinese public health policy should also pay attention to those with high levels of education attainments, who are expected to live additional time with a disability, and probably expend more on health and long-term care. Once disability occurs, the probability of recovery is slight over the duration of 3 years and does not vary between groups with different educational attainments. More resources therefore should be focussed on health maintenance in earlier life stages before the onset of disability, which calls for a comprehensive and feasible disability prevention strategy. Public policies aiming to improve accessibility of health services, reduce income or wealth inequality, and promote healthy lifestyles could help reduce education-related disparities in functional health status transition and enhance healthy ageing among older persons.

## Abbreviations

ADLs: Activities of Daily Livings
AIC and BIC: The Akaike and Bayesian information criteria
BMI: Body mass index
CLHLS: Chinese Longitudinal Healthy Longevity Survey
GSEM: Generalized structural equation model
